# Editorial: Early child development in play and education: A cultural-historical paradigm

**DOI:** 10.3389/fpsyg.2022.968473

**Published:** 2022-07-12

**Authors:** Nikolay Veraksa, Ingrid Pramling Samuelsson, Yeshe Colliver

**Affiliations:** ^1^Department of Educational Psychology and Pedagogy, Faculty of Psychology, Lomonosov Moscow State University, Moscow, Russia; ^2^Department of Education, Communication and Learning, Gothenburg University, Gothenburg, Sweden; ^3^Department of Educational Studies, Faculty of Human Sciences, Macquarie University, Sydney, NSW, Australia

**Keywords:** guided play, executive functions (EF), home learning environment (HLE), free play, language development, playful learning, teachers' perspectives

## Introduction

The current Research Topic considers some of the latest research on early development in relation to the cultural-historical theory of Vygotsky (1996), who considered education and play in the context of child development and learning. In particular, Vygotsky singled out a form of education that focused not on already established cognitive structures, but on those that are emerging (Vygotsky, 1991). In this context, Vygotsky spoke of learning as an educational activity because it generates a zone of proximal development, a zone in which the child operates at the edge of her emerging abilities and approaches the expert cultural knowledge of the adult. For Vygotsky, the zone of proximal development is an important educator's tool for effectively influencing not only learning but even the formation of children's consciousness. It is particularly pertinent for early education as the emergent properties of the zone are evident not only in the process of instruction, but also of play, which forms the basis of early curricula around the developed world (Organization for Economic Cooperation Development, 2017). Of particular focus in the articles is the role of the adult in supporting this zone of proximal development as they engage in guided play with children, a topic of much recent research attention and scholarly debate (e.g., Weisberg et al., 2016; Eason and Ramani, 2020; Veraksa et al., 2021). The articles of this Research Topic present the latest research on the possibilities of child development in play and learning, with particular focus on the dialectical relationship between individual creativity and social conformity.

## Development in play

In the first article in the Research Topic, Cai et al. tested the hypothesis that an intervention based on the use of a thematic block play would positively affect the language development of children. An essential point of the intervention was group discussions about the construction projects. In the control group, children played blocks on their own without the participation of adults. The authors propose three reasons for the results supporting the hypothesis: creating a learning context that allowed children to express their thoughts and feelings; high motivation due to the children's interest in the theme; and egalitarian communication between teachers and children.

In their Section Discussion, Cai et al. (p. 6) identify the key factor in the influence of thematic play with blocks as the fact that the intervention group, in comparison with the children of the control group, had significantly greater opportunities for describing components of play such as an imaginary situation, roles, the plot, the rules, etc. In this case, the play situation appears to have been the basis for the construction of the zone of proximal development under the guidance of an adult. The actions of the adult allowed the children not only to discuss the construction of structures in a verbal plan, but also to create an imaginary situation and use the imaginary situation to the fullest to develop their abilities. In contrast, the play activity of preschoolers in the control group was not as developed.

Similar conclusions are reached by the Research Topic reported by Veresov et al. The authors single out the role of adult support in play's essential attributes: the presence of an imaginary situation, the assumption of a role, and action in accordance with a set of rules determined by the role and plot. Their research was consistent with play creating a zone of proximal development, in which adequate adult guidance acting as a training space for the development of executive functions. Thus, working memory makes it possible to retain role rules and values of substitute objects; inhibitory control restrains impulsive actions in accordance with the role played; and cognitive flexibility allows children to move back and forth from a real to an imaginary situation. This movement within the zone also allows children to develop individual creative ideas but also balance them within social rule conformity.

To a certain extent similar results are obtained in the article by Lin et al. In order to achieve positive academic results for children, Chinese educators prefer to support adult-led play rather than children-led play. This is identified as partly due to the influence of the Confucian philosophical tradition that attaches great importance to adult instruction. According to Vygotsky's point of view, the development of a child is conditioned by the processes of transformation of natural functions into higher mental functions, and this applies to both academic as well as socio-emotional learning. In contrast, only a small minority of Chinese educators showed a valuing of children learning socio-emotional skills *via* free play, with the vast majority valuing academic learning through guided play. Although empirical support remains larger for the former than the latter (Zosh et al., 2018), meticulous development of policy and curriculum is needed to ensure educators' views on play are a support rather than hindrance for the implementation of a play-based curriculum.

In a more direct approach Veraksa et al. sought to test Vygotsky's assumption that play does develop with preschool children's executive function development. While it should be noted that the characteristics of higher mental functions proposed by Vygotsky differ from the executive functions' indicators used in the study, the results indicate interesting patterns wherein play leadership and rule conformity, but not preferences, are related to executive functions. This research in highlights the relative importance of inspiring others with one's own play proposals (creativity), as well as conformity to (social) rules, as central mechanisms to the use and development of executive functions, adding to the growing empirical explorations of Vygotsky's theories on the relationship between play and executive functions.

The article by Della Porta et al. analyzes the process of naturalistic parenting at home, highlighting the importance of rule conformity in particular. From Vygotsky's (1983) general genetic law of cultural development, which states that any function “in the cultural development of the child appears on the stage twice, on two planes, first social, then psychological, first between people, as an interpsychic category, then inside the child, as an intrapsychic category” (p. 145), the possibility of natural home teaching follows. This process is multifaceted. Parents teach children a wide range of cultural skills in a variety of settings, including situations of play and conflict. Such teaching sets the stage for the creation of the zone of proximal development. This establishment of the social plane for children is evidenced in the data from Della Porta et al., where mothers were shown to engage in more social rule disposition teaching than fathers, and fathers more game rule knowledge than mothers. This research underscores the importance of the sociocultural role that both parents take in the remit of home culture, particularly in playful interactions, but particularly underscore the creation of the zone of proximal development as parents engage with children at their actual developmental level and tie this to the social plane by setting cultural expectations *via* social rules.

## Conclusion

These papers support the emerging research on the role of the adult in guiding play toward optimal development. In our opinion, there are three forms in which development is realized in preschool age: learning, play, and creativity. If learning is largely determined by adults, then play, depending on the position of the adult (from complete leadership to non-intervention), supports either its cultural (learned) or natural (innate) aspects (Veraksa et al., 2021). At the same time, culture to a lesser or greater extent retains its influence in play through the process of imitation (particularly in pretend play). In play, creativity is the process of preschoolers implementing their ideas, which also supports their development, since development is contingent on the emergence of new ideas. Thus, it is necessary to keep in mind the three forms of development and the corresponding roles of the adult for these processes. These roles provide some explanation for the results presented in this Research Topic, as well as routes for future research.

## Conflict of interest

The authors declare that the research was conducted in the absence of any commercial or financial relationships that could be construed as a potential conflict of interest.

## Author Contributions

All authors listed have made a substantial, direct, and intellectual contribution to the work and approved it for publication.

## Publisher's Note

All claims expressed in this article are solely those of the authors and do not necessarily represent those of their affiliated organizations, or those of the publisher, the editors and the reviewers. Any product that may be evaluated in this article, or claim that may be made by its manufacturer, is not guaranteed or endorsed by the publisher.
